# Efficient derivation of chimeric-antigen receptor-modified T_SCM_ cells

**DOI:** 10.3389/fimmu.2022.877682

**Published:** 2022-07-28

**Authors:** Emiko Kranz, Charles J. Kuhlmann, Joshua Chan, Patrick Y. Kim, Irvin S. Y. Chen, Masakazu Kamata

**Affiliations:** ^1^ Division of Hematology-Oncology, David Geffen School of Medicine at University of California, Los Angeles (UCLA), Los Angeles, CA, United States; ^2^ Department of Microbiology, School of Medicine, University of Alabama at Birmingham, Birmingham, AL, United States; ^3^ Department of Microbiology, Immunology, and Molecular Genetics, David Geffen School of Medicine at University of California, Los Angeles (UCLA), Los Angeles, CA, United States

**Keywords:** T_SCM_, CAR, gene therapy, adoptive immunotherapy, HIV-1

## Abstract

Chimeric-antigen receptor (CAR) T-cell immunotherapy employs autologous-T cells modified with an antigen-specific CAR. Current CAR-T manufacturing processes tend to yield products dominated by effector T cells and relatively small proportions of long-lived memory T cells. Those few cells are a so-called stem cell memory T (T_SCM_) subset, which express naïve T-cell markers and are capable of self-renewal and oligopotent differentiation into effector phenotypes. Increasing the proportion of this subset may lead to more effective therapies by improving CAR-T persistence; however, there is currently no standardized protocol for the effective generation of CAR-T_SCM_ cells. Here we present a simplified protocol enabling efficient derivation of gene-modified T_SCM_ cells: Stimulation of naïve CD8+ T cells with only soluble anti-CD3 antibody and culture with IL-7 and IL-15 was sufficient for derivation of CD8+ T cells harboring T_SCM_ phenotypes and oligopotent capabilities. These *in-vitro* expanded T_SCM_ cells were engineered with CARs targeting the HIV-1 envelope protein as well as the CD19 molecule and demonstrated effector activity both *in vitro* and in a xenograft mouse model. This simple protocol for the derivation of CAR-T_SCM_ cells may facilitate improved adoptive immunotherapy.

## Introduction

Gene-engineered T cells with desired antigen-specific receptors such as chimeric-antigen receptors (CARs), aim to confer directed and enhanced cytotoxic T lymphocyte responses (1–4). CARs contain an antigen-binding domain specific for targets and an internal-signaling domain derived from CD3ζ chain as well as 41BB, CD28, or other co-stimulatory molecules (5, 6). When a CAR encounters its target ligand, it signals the cell in a T cell receptor-like, but human-leukocyte antigen (HLA)-independent manner, thus allowing this approach to be used in any patient. In human clinical trials for B-cell malignancies, CAR-modified autologous T cells targeting CD19 have shown robust effector responses (5–10). For HIV-1 infection, CD4ζ CAR has been widely and longitudinally tested in patients. Treatment was safe and well-tolerated for over a decade, but antiviral effects were limited due to low levels of gene-modified cell persistence (11–14).

The novel memory T cell subset known as stem cell-memory T (T_SCM_) harbors self-renewing and oligopotency capabilities. The T_SCM_ subset has been identified in mice (15), non-human primates (16, 17), and humans (18, 19). In humans, approximately 2-4% of peripheral blood T cells consist of T_SCM_ cells (20). The T_SCM_ cells are phenotypically defined as naïve T (T_N_) cells by the expression of T_N_ cell markers, such as CD45RA, CD62L and CCR7, but are distinguishable from T_N_ cells by two memory T cell markers: CD95 and CD122. Thus, they exhibit a gene profile between T_N_ and central-memory T (T_CM_) cells. Of note, the T_SCM_ cells ― unlike other memory T cells ― can be expanded *ex vivo* while maintaining their stemness, allowing enrichment of the gene-modified population prior to transplant.

Increasing evidence indicates that the T_SCM_ cells exhibit a lesser extent of proliferation and effector activities compared to other memory T cells. Upon antigen stimulation, the T_SCM_ cells have the ability to differentiate into T_CM_ (which are thought to be a premature memory subset that differentiates into the effector subset upon antigen re-encounter), followed by effector-memory T (T_EM_) cells (which are considered to be committed progenitor cells that undergo terminal differentiation after a limited number of cell divisions) (18, 21–24). The frequency and activity of T_SCM_ cells from clinical samples also supports their prolonged precursor potential in humans (25, 26). Importantly, individual gene-modified T_SCM_ cell clones did not show the emergence of clonal dominance for over a decade after infusion, indicating that gene engineering of T_SCM_ cells does not bring in them a tumorigenic change. These evidence well support that T_SCM_ cells would be an ideal host cell for CAR engineering.

We here developed a simple condition for the derivation and expansion of gene-modified-T cells harboring T_SCM_-surface phenotype and validated its applicability for gene engineering of T cells using both anti-HIV-1 and anti-CD19 CARs. Our results further demonstrate that these cells can effectively differentiate to functional-T cells conferring CAR-dependent effector activity against target cells *in vitro* as well as in a xenograft NSG mouse model.

## Methods

### Cells

**Figure 1:**
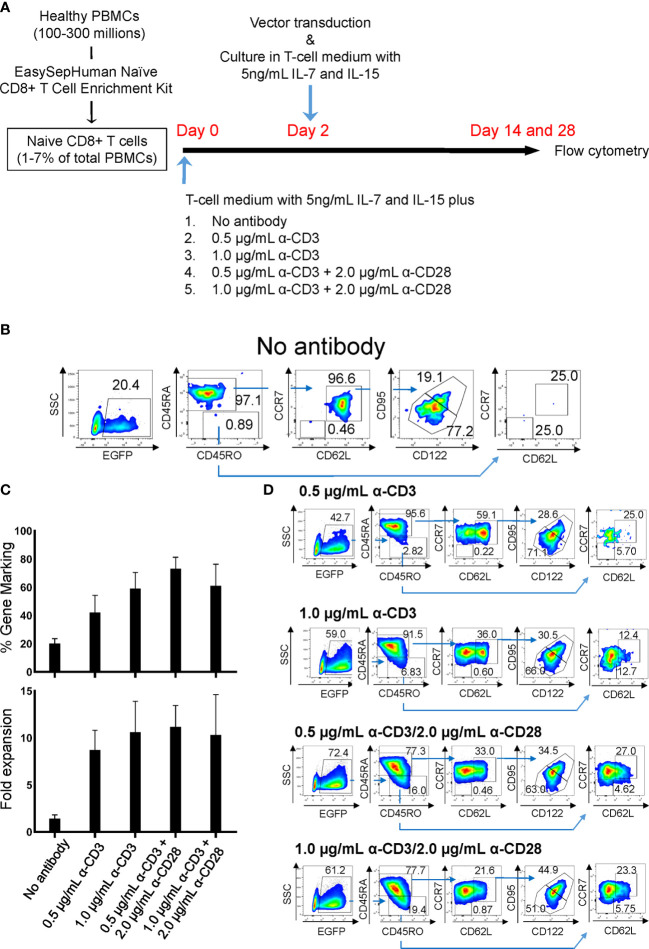
Derivation of gene-marked CD8+ T cells harboring T_SCM_-surface phenotype under different stimulation conditions. Freshly isolated human PBMCs were separated for CD8+ T_N_ cells using an EasySep™ Human Naïve CD8+ T Cell Enrichment kit. Cells were stimulated for 2 days with the condition as in **(A)**, followed by transduction with lentiviral vector encoding EGFP as a transduction marker. Cells were cultured for an additional 12 days in the presence of 5 ng/mL of IL-7 and IL-15, and cell-surface marker profiles were analyzed by flow cytometry. **(A)** Summary for derivation of T_SCM_ cells procedure. **(B)** Flow cytometry analyses of CD8+ T_N_ cells genetically marked by EGFP with no antibody stimulation. **(C)** % positivity of EGFP-marked cells (Top bars) and fold changes in cell number following 14 days of culture (Bottom bars). **(D)** Flow cytometry analyses of EGFP-engineered CD8+ T_N_ cells. All experiments were repeated at least three times. Error bars in **(C)** show the standard deviation of a data set. One representative experiment is shown for **(B, D)**.

Peripheral blood mononuclear cells (PBMCs) from healthy human donors were obtained from the CFAR Virology Core at UCLA without personal identifying information. To minimize a potential induction of T-cell stimulation by events such as crosslinking of cell-surface molecules, T_N_-cell population was negatively selected in one step using an EasySep™ Human Naive CD8+ T Cell Enrichment Kit (StemCell Technologies, Inc., Vancouver, Canada), which consistently provides >95% purity of CD8+ T_N_ cells (**Supplementary Figure 1**). Cells were maintained in Iscove’s Modified Dulbecco’s Medium (IMDM) supplemented with 1% GlutaMAX supplement and Antibiotic-Antimycotic (Life Technologies, Grand Island, NY), 20% FCS (SH30070.03E; GE Healthcare Life Sciences, South Logan, UT), and 0.1 mM 2-mercaptoethanol (Sigma-Aldrich St. Louis, MO) (T-cell medium) as reported previously (27). Prior to lentiviral vector transduction, CD8+ T_N_ cells were incubated with various concentrations of anti-CD3 antibody (Hit3a; BioLegend, San Diego, CA) with or without 2 µg/mL of anti-CD28 antibodies (CD28.2; BioLegend) for 2 days in T-cell medium supplemented with 5 ng/mL IL-7 and IL-15 (R&D systems, Minneapolis, MN) as summarized in **Figure 1A**. All cells were incubated at 37°C in 5% CO_2_.

### Cytotoxic assay

Triple-CD4ζ (27) was used an anti-HIV CAR. For anti-HIV CAR cytotoxicity assays, Jurkat cells encoded without (ΔKS, non-target cells) or with HIV-1_HXBC2_ envelope protein (HXBC2, target cells), where the expression can be induced by removal of doxycycline from culture medium (28), were used. As an additional target cell for *in vivo* cytotoxicity assay, TF228.1.16 cells ― which are BJAB cells constitutively expressing HIV-1_BH10_ envelope protein (29) ― were used. ΔKS, HXBC2, and TF228.1.16 cells were genetically labeled with TagBFP (30), mCherry (31), and mStrawberry (31), respectively. In an assay for anti-CD19 CAR (FMC63-IgG4ζ (32)), we used BCBL-1 (33) (CD19-, non-target cells) and Ramos cells (CD19+, target cells). BCBL-1 and Ramos cells were genetically labeled with TagBFP and mStrawberry, respectively. All cells were obtained from the NIH-AIDS Reagent Program and cultured as recommended.

For *in vitro* cytotoxicity assay, 5 x 10^4^ CAR- or EGFP-modified CD8+ T cells as a negative control were plated using 100µL of T-cell medium in a 96-well round bottom plate. The same numbers of genetically-labeled ΔKS and HXBC2 cells for CD4ζ or BCBL-1 and Ramos cells for FMC63-IgG4ζ were added to each well and co-incubated for 4 or 16 hours [Effector: Target (E:T) ratio = 1:1]. Antigen specificity of both CARs was also validated with HXBC2 cells culturing with doxycycline to suppress HIV-1_HXBC2_ envelope protein expression for CD4ζ CAR which cells cannot be a target for CD4ζ CAR, as well as human CD19 overexpressing HXBC2 cells culturing with doxycycline for FMC63-IgG4ζ CAR (data not shown). Total numbers of each cell were determined by MACSQuant (Milteny Biotech, Germany) and relative cytotoxicity of target cells relative to non-target cells was determined by the following formula: Relative cytotoxicity (%) =100 x (1 – target cell number/non-target cell number).

### Viruses

Lentiviral vectors were generated and transduced as described elsewhere (27, 34, 35). p24^Gag^ ELISA assays were performed by the CFAR Virology Core at UCLA. Lentiviral vector information used in this research will be provided upon request.

### Flow cytometry

The following antibodies were used in flow cytometry: BV711-CD4 (OKT4), BV605-CD8 (RPA-T8), APC/Cy7-CD45RO (UCHL1), AlexaFlour700-CD45RA (H100), PerCP/Cy5.5-CD62L (DREG-56), BV785-CCR7 (G043H7) (all from BioLegend), eFluor650NC-CD3 (OKT3; eBioscience, San Diego, CA), APC-CD95 (LT95; Thermo Fisher, Pittsburgh, PA), and BV421-CD122 (Mik-β3; BD Biosciences, San Jose, CA). Cells were acquired using FACSDiva on BD LSRFortessa. Data for each cell with different cell surface phenotypes were analyzed using FlowJo software (BD Biosciences) as summarized in **Supplementary Figure 2**. Absolute cell counts were determined using MACSQuant analyzer. Cell sorting was performed by the CFAR Flow Cytometry Core Facility at UCLA.

### *In vivo* tumor-killing assay

Animal research was conducted under UCLA’s Chancellor’s Animal Research Committee. Two million of TF228.1.16 or HXBC2 cells in 50 μL of PBS were mixed with 50 μL of Matrigel (BD Biosciences) and subcutaneously engrafted to the left or right hind limbs of NOD.CB17-Prkdc*^scid^*/J (NOD-SCID) mice (n=4) (Jackson Laboratory, Bar Harbor, Maine), respectively. On day 14, either Triple-CD4ζ- or FMC63-IgG4ζ–modified T cells (5x10^5^) were infused *via* the retro-orbital vein. Biofluorescence images and the weight of xenograft tumors were obtained 42 days post-engraftment.

### Statistical analyses

Results are expressed as mean ± standard deviations (SDs). Errors depict SD. Comparisons between two groups were performed using an unpaired two-tailed t-test with Welch’s correction. A *p*-value less than 0.05 was considered statistically significant.

## Results

### Stimulation of CD8+ T_N_ cells with soluble anti-CD3 antibody enables efficient gene-modification by lentiviral vector while minimizing T-cell differentiation

**Figure 2:**
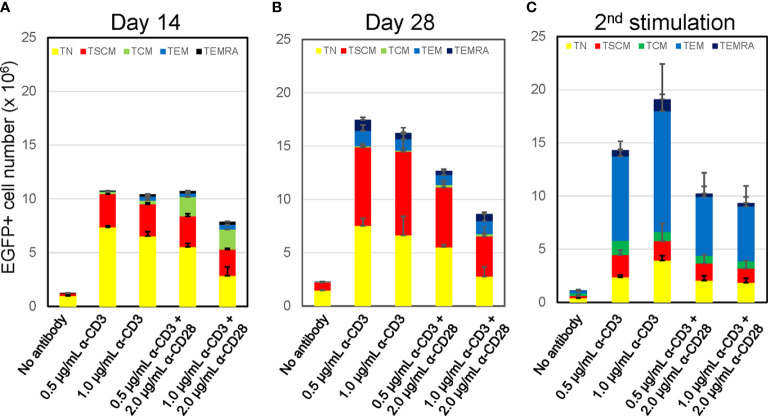
Prolonged culture increases % population of CD8+ T cells harboring T_SCM_-surface phenotype with maintaining oligopotency Freshly isolated CD8+ T_N_ cells were transduced with lentiviral vector encoding EGFP following stimulation with various antibody conditions shown in **Figure 1A**. The cells were cultured for an additional 12 days (**A**, Day14) or 26 days (**B**, Day 28) in the presence of 5 ng/mL of IL-7 and IL-15. A half million of cells in A was co-stimulated by 0.5 μg/mL anti-CD3 and 2.0 μg/mL anti-CD28 antibodies and further cultures for 14 days (**C**, 2^nd^ stimulation). Cells were staining for CD45RA, CD45RO, CCR7, CD62L, CD95 and CD122, and surface marker of EGFP-marked cells were analyzed by flow cytometry. Each cell number of EGFP-marked cells with T_N_, T_SCM_, T_CM_, T_EM_, and T_EMRA_ phenotypes was plotted. Experiments were repeated three times. Error bars show the standard deviation of a data set.

Two independent protocols for the derivation of T_SCM_ have been described by Gattinoni et al. (18) and Cieri et al. (19). The major differences between those two protocols are that the former used an inhibitor of glycogen synthase kinase 3β (GSK-3β), TWS119 (15, 36), and the latter used a low concentration of two cytokines, IL-7 and IL-15 with no chemical inhibitor. Our preliminary experiments for a side-by-side comparison of those two protocols showed that there was minimal benefit of the use of TWS119 for the derivation of T_SCM_ cells due to a poor cell expansion by the former protocol. Whereas, the latter protocol was able to yield a nearly 5-fold higher number of cells harboring surface phenotypes corresponding to T_SCM_ (0.41 x 10^6^ *vs* 1.88 x 10^6^, **Supplementary Table 1**). However, a considerable number of T_EM_ cells were derivated under both conditions, presumably due to the potent stimulation condition of using anti-CD3/CD28 antibody coated beads. We thus assessed alternative stimulation conditions for their ability to derive large numbers of T_SCM_ while minimizing T-cell differentiation and allowing for gene modification *via* lentiviral vectors. Negatively selected CD8+ T_N_ cells were incubated with different amounts of soluble anti-CD3 antibody with or without anti-CD28 antibody. The cells were then transduced with a lentiviral vector encoding EGFP as a marker gene and cultured for an additional 12 days with IL-7 and IL-15 followed with Cieri’s protocol (**Figure 1A**). In the no antibody condition, approximately 20% of CD8+ T_N_ cells were successfully gene-modified, indicating that a small portion of CD8+ T cells are transducable without active stimulation and that culturing in the presence of IL-7 and IL-15 allows maintenance of the CD8+ T_N_ cell phenotype for at least 14 days (**Figure 1B**). However, the no antibody condition resulted in extremely poor expansion of the cells (<2-fold, **Figure 1C**). The inclusion of increasing concentrations of anti-CD3 antibody and/or the presence of anti-CD28 antibody increased the levels of gene marking and T-cell expansion (**Figure 1C**), but also increased the proportion of differentiated cells as defined by the loss of CD45RA or CD62L expression (37–39) (**Figures 1D**, **2A**). The differences shown above became more obvious after an additional 14 days of culture (total 28 days of culture) (**Figure 2B**, Day 28), indicating that cells harboring T_SCM_-surface phenotype slowly but continuously expanded in the presence of IL-7 and IL-15. The average numbers from each population in EGFP-positive cells after a total of 28 days of culture in **Figure 2B** were summarized in **Supplementary Table 2**: a higher recovery of the EGFP-marked T_SCM_ cells was observed with 0.5 µg (7.35 x 10^6^) or 1.0 µg/mL anti-CD3 (7.84 x 10^6^) stimulation rather than the condition with 0.5 µg/mL anti-CD3 + 2.0 µg of anti-CD28 antibody (5.61 x 10^6^), which generated the highest gene marking as in **Figure 1C**. Importantly, EGFP-marked T_SCM_ cells were expanded nearly 2-fold following a total of 28 days of culture, whereas the number of T_SCM_ cells in other populations decreased over the course of the culture (**Supplementary Figure 3**). These results indicate that stimulation of CD8+ T_N_ cells with only soluble anti-CD3 antibody (0.5-1.0 µg/mL) and prolonged-expansion (28 days) in the presence of IL-7 and IL-15 allowed for the most efficient derivation of gene-marked CD8+ T cells harboring T_SCM_-surface phenotype with a minimum level of T-cell differentiation.

A key feature of the T_SCM_ subset is its oligopotency allowing differentiation into more terminal subsets upon antigen stimulation (18, 19). We confirmed the differentiation ability of these *in-vitro* generated T_SCM_ cells *via* anti-CD3/CD28 co-stimulation. The status of T-cell differentiation was evaluated by surface phenotypes at 14-days after the 2^nd^ stimulation (**Figure 2C**, 2^nd^ stimulation). The number and prevalence of CD8+ T cells harboring the T_SCM_ and T_N_ phenotype was substantially reduced after the 2^nd^ stimulation. Concomitantly, the proportion of differentiated cells ─ especially the T_EM_ population ─ increased under this condition. Thus, these *in-vitro* generated T_SCM_ cells retain their oligopotency.

### Derivation of CAR-modified CD8+ T cells with T_SCM_-surface phenotype

**Figure 3:**
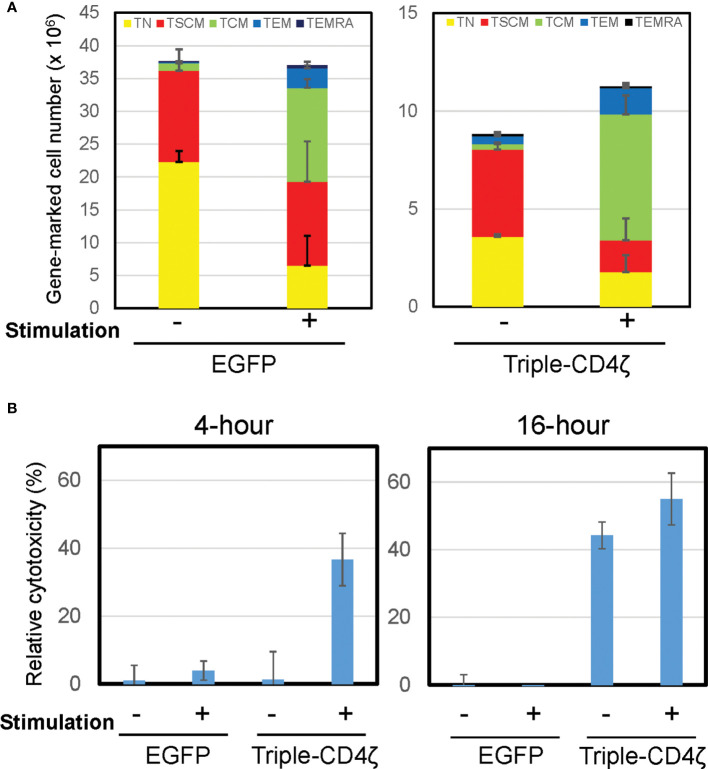
Induced differentiation of Triple-CD4ζ modified CD8+ T cells harboring T_SCM_-surface phenotype with anti-CD3 and CD28 co-stimulation. **(A)** Triple-CD4ζ or EGFP-modified CD8+ T cells were co-stimulated with 0.5 µg/mL of anti-CD3 and 2.0 µg/mL of CD28 antibodies for 2 days (2^nd^ stimulation) at day14 post-1^st^ stimulation. The cells were further cultured for an additional 12 days in the presence of 5 ng/mL of IL-7 and IL-15. Cell-surface profiles of gene-marked CD8+ T cells were analyzed by flow cytometry. Each cell number of EGFP- or Triple-CD4ζ-marked cells with T_N_, T_SCM_, T_CM_, T_EM_, and T_EMRA_ phenotypes was plotted with or without 2^nd^ stimulation. Experiments were repeated three times. Error bars show the standard deviation of a data set. **(B)** The cells were plated at 5 x 10^4^ cells/100 μL in a 96-well plate. The same number of TagBFP-labeled Jurkat cells (ΔKS, non-target control) and mCherry-labeled Jurkat cells constitutively expressing HIV-1_HXBC2_ envelope protein (HXBC2, target cells) were added to the wells and incubated for 4 or 16 hours. Total numbers of each population were determined by MACSQuant, and relative cytotoxicity of target cells relative to non-target cells was determined. -: unstimulated, +: 2^nd^ stimulated. Experiments were repeated three times. Error bars show the standard deviation of a data set. Cytotoxicity assays were performed in biological triplicate.

As above, we were able to validate the oligopotency of the *in-vitro* generated gene-engineered cells with T_SCM_-surface phenotypes. We next applied this method for the generation of anti-HIV-1 CAR-T cells. We utilized the Triple-CD4ζ CAR that targets HIV-1 gp120 on the infected cell surface (27) with co-expression of two anti-HIV-1 shRNAs: sh1005 which suppresses surface expression of the key HIV-1 co-receptor CCR5 (40) and sh516 which prevents HIV-1 infection to both CD4+ T and CD8+ T cells mediated *via* CD4ζ expression (34). Thus, the Triple-CD4ζ CAR can exert potent anti-HIV-1-effector activity while protecting its transduced cells from HIV-1 infection. We previously demonstrated its potent anti-HIV-1 effects *in vivo* using an HIV-1 challenged-humanized mouse model (41, 41). Although the condition established above allowed successful modification of CD8+ T_N_ cells with the Triple-CD4ζ CAR while maintaining a population with the T_SCM_-surface phenotype, the proportion of transduced cells was relatively lower compared to that of control EGFP vector (42.9% *vs* 8.8%, **Supplementary Figure 4**). This is potentially due to a larger size of the transgene (42). Nevertheless, these gene-marked cells were successfully differentiated to cells harboring surface phenotypes of T_CM_ and T_EM_ cells upon anti-CD3/CD28 stimulation (**Figure 3A**).

We next assessed the effector function of Triple-CD4ζ-modified CD8+ T cells by a flow-cytometry based cytotoxicity assay. The cells modified with Triple-CD4ζ cells were incubated with their target Jurkat cells expressing HIV-1_HXBC2_ envelope protein (HXBC2) upon doxycycline removal from culture medium (28). To validate the level of non-specific target cell killing, control Jurkat cells which do not encode HIV-1-envelope protein (ΔKS, non-target cell) were included (28). The former is labeled by mCherry and the latter by Tag-BFP, thus both cells can be distinguished by flow cytometry. The numbers of remaining mCherry+ and TagBFP+ cells following incubation with Triple-CD4ζ-modified CD8+ T cells correspond to levels of anti-HIV-1 CAR-dependent and independent cytotoxicity, respectively. Following a co-culture with the above three cells for 4 and 16 hours, the total numbers of mCherry+ and TagBFP+ cells were analyzed by MACSQuant, and relative cytotoxicity against target cells compared to that against non-target cells was determined (**Figure 3B**). The pre-differentiated cells shown in **Figure 3A** were used as a positive control for the assay. There were minimal levels of change in the cell surface phenotypes of Triple-CD4ζ following 4-hour and 16-hour incubations with the target cells (**Supplementary Figure 5**). A 4-hour incubation was sufficient for the induction of cytotoxic activity by the pre-differentiated cells transduced with Triple-CD4ζ, whereas a 16-hour incubation was required to achieve a similar level of cytotoxicity without pre-differentiation, indicating that longer incubation was required to exert an effector activity for undifferentiated Triple-CD4ζ-modified CD8+ T cells.

Additionally, we generated anti-CD19 (FMC63-IgG4ζ) modified CD8+ T cells using the same conditions as above (**Supplementary Figure 6**). The level of gene marking by FMC63-IgG4ζ was similarly lower than control vector as seen with Triple-CD4ζ. FMC63-IgG4ζ-modified cells also maintained high levels of expression for T_SCM_ cell markers, and a large portion of the cells harbored T_N_- and T_SCM_-surface phenotypes. Similar to Triple-CD4ζ modified-CD8+ T cells, FMC63-IgG4ζ-modified cells also exerted a potent antigen dependent cytotoxicity following 16, but not 4, hours of incubation.

### Triple-CD4ζ CAR-modified CD8+ T cells generated with sole anti-CD3 antibody stimulation exert antigen-specific effector functions in a xenograft NOD-SCID mouse model

As above, CD8+ CAR-T cells carrying the T_SCM_-surface phenotype were generated *in vitro* with an antigen-specific effector activity. We next tested their effector activity *in vivo* using a xenograft NOD-SCID mouse model. NOD-SCID mice were engrafted with two different lymphoma cell lines: TF228.1.16 ― a derivative of BJAB (29) or HXBC2 ― a derivative of Jurkat (28). These lines express envelope protein from either the HIV-1_BH10_ or HIV-1_HXBC2_ strains, respectively. *In vitro*, the TF228.1.16 cells were killed by Triple-CD4ζ-modified CD8+ T cells like the HXBC2 cells, but relatively weakly by FMC63-IgG4ζ-modified CD8+ T cells. Although TF228.1.16 is CD19+ and can be a target for FMC63-IgG4ζ-modified CD8+ T cells *in vitro* (**Supplementary Figure 7**), our FMC63-IgG4ζ construct contains an IgG hinge-CH2-CH3 domain, which makes ineffective this CAR *in vivo* due to an Fcγ receptor 1 mediated CAR-T cell elimination (43–46). Thus, the FMC63-IgG4ζ serves as negative control. Those two tumor cells were genetically marked by mStrawberry (TF228.1.16) or mCherry (HXBC2), and subcutaneously engrafted into the left and right hind limbs of NOD-SCID mice, respectively. We confirmed that the expression levels of HIV-1 envelope proteins in tumor tissues developed in xenograft mice were similar to those in cells maintained *in vitro* by western blotting using anti-GP120 (2G12) and anti-GP41 (2F5) antibodies (data not shown).

**Figure 4:**
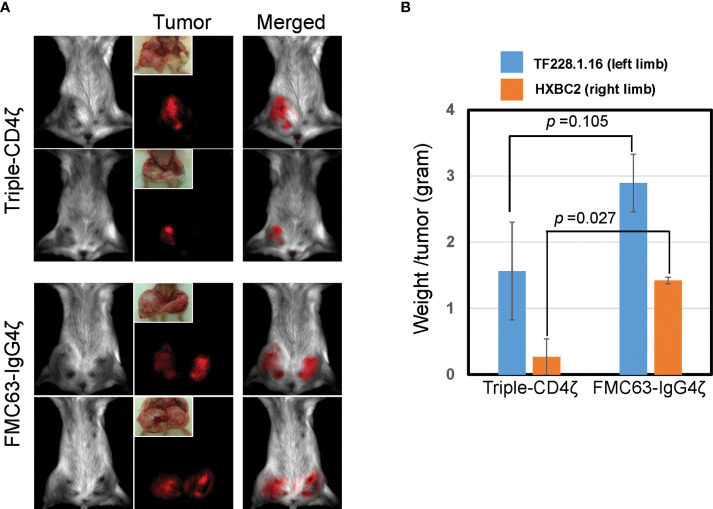
Triple-CD4ζ CAR-modified CD8+ T cells harboring T_SCM_-surface phenotype eliminate tumor cells expressing HIV-1 envelope proteins in a xenograft mouse model. Two million mStrawberry-labeled CD19+ TF228.1.16 cells expressing envelope protein from HIV-1_BH10_ or mCherry-labeled Jurkat cells expressing envelope protein from HIV-1_HXBC2_ (HXBC2), were mixed with Matrigel at a 1:1 ratio and subcutaneously engrafted to the left or right hind limbs of NOD-SCID mice from ventral side, respectively (n = 4). Freshly isolated CD8+ T_N_ cells were stimulated for 2 days with 0.5 µg/mL of anti-CD3 antibody in T-cell medium, followed by transduction with a lentiviral vector encoding either Triple-CD4ζ or FMC63-IgG4ζ. Following 26 days of culture in the presence of 5 ng/mL of IL-7 and IL-15, cells corresponding to 5 x 10^5^ CAR-modified cells were intravenously injected *via* the retro-orbital vein on day 14 post-engraftment of TF228.1.16 and HXBC2. Biofluorescence images **(A)** and the weight of xenograft tumors **(B)** were obtained on day 42 post-engraftment (on day 28 post-transplant of CAR cells). **(B)** Blue or orange bars: average weight of xenograft tumors from TF228.1.16 (left limb, blue bars) or HXBC2 (right limb, orange bars) on day 42 post-engraftment. Experiments were repeated three times with similar results. Two representative animals from each group were shown.

At 14 days post-engraftment, we infused CD8+ T cells engineered with either Triple-CD4ζ CAR or FMC63-IgG4ζ CAR derived as above. We analyzed these CAR-dependent effector activities by bioimaging using a Maestro 2 multispectral imaging system (**Figure 4**). As expected, we observed poor *in vivo* effector activity of the cells modified by FMC63-IgG4ζ CAR. On the other hand, the cells modified by Triple-CD4ζ CAR showed stronger anti-tumor effector activity against both tumors, with tumor burdens decreasing approximately 1.9-fold for TF228.1.16 tumors (blue bars, **Figure 4B**) and 5.5-fold for HXBC2 tumors (orange bars, **Figure 4B**). Compared to HXBC2 tumor, the effector activity on TF228.1.16 tumor was weaker with no statistical significance; this may be due to the faster growth rate of TF228.1.16 cells. These results provide additional evidence that our simplified protocol allows for the derivation of CAR-modified CD8+ T cells harboring T_SCM_-surface phenotype while maintaining antigen-specific effector activity *in vivo*.

## Discussion

Though there is currently no standardized protocol for CAR-T_SCM_ cell manufacturing, the use of CAR-T_SCM_ products represents a promising approach for improving the outcome of CAR-T based therapies. Here, we refined the protocol for the derivation of CAR-modified CD8+ T cells harboring T_SCM_-surface phenotype and T_SCM_-like oligopotency.

The first key to generating CAR-T_SCM_-like cells is the purification of CD8+ T_N_ cells as a starting population. Flow-cytometry based cell sorting is commonly used to isolate this population, but has 2 major drawbacks: decreased cell viability (47, 48), and the potential for sorting antibodies to induce unwanted activation *via* binding to T_N_ cell surface markers. By this reasoning, negative selection may be more effective for isolating T_N_ populations because these processes leave the cells ‘untouched’ and also without any antibodies remaining bound to the cells in the final product. By using an EasySep™ Human Naïve CD8+ T Cell Enrichment Kit which enables negative selection of CD8+ T_N_ cells, we consistently obtained CD8+ T_N_ cells with >95% purity (**Supplementary Figure 1**).

Next, we evaluated the utility of different stimulation conditions for generating gene-modified T_SCM_-like CD8+ T cells and found that stimulation with only soluble anti-CD3 antibody in the presence of IL-7 and IL-15 was the most efficient. Since this condition does not require the use of novel agents, it is readily translatable for clinical applications. Cieri *et al.* also tested a stimulation condition with only anti-CD3 antibody for expansion of cells with a CD8+/CD45RA+/CD62L+/CD95- phenotype, but this attempt was unsuccessful (19). There were several differences compared to our protocol; they used a different anti-CD3 antibody clone ― OKT3 ― at >16-times lower concentration (30 ng/mL) in a plate-bound format. With this condition, they observed a poor expansion of CD8+ T_N_ cells compared to the cells co-stimulated with anti-CD3/CD28 antibody coated beads. Surprisingly, most of the cells lost expression of CD45RA and CD62L after 16 days of culture with their experimental conditions (19). We did not observe this by the use of soluble form of anti-CD3 antibody, suggesting that the precoated anti-CD3 antibodies probably induced a stronger stimulation *via* TCR crosslinking, resulting in T-cell differentiation.

Whereas both CD4+ and CD8+ T cells are needed for an efficient CAR-T cell therapy (49–51); the former mainly serve as CAR-dependent helper cells and the latter as CAR-dependent effector cells (52). We have already adopted the derivation of CAR-modified CD4+ T_SCM_ cells by the use of EasySep Human Naive CD4+ T cell Isolation kit, which enables negative selection of CD4+ T_N_ cells (Stemcell technologies, #19555). Unlike CD8+ T_N_ cells, CD4+ T_N_ cells required co-stimulation by soluble forms of anti-CD3 (1.0 μg/mL) and anti-CD28 (0.1 μg/mL) antibodies for an efficient expansion of cells with T_SCM_-surface phenotype (data not shown). The level of gene marking as well as oligopotential activity of CAR-engineered CD4+ T_SCM_ cells were similarly reproduced as those confirmed in CAR-engineered CD8+ T_SCM_ cells. We are currently validating the effector activity of CAR-engineered CD4+ T cells as well as seeking to derivate CAR-engineered T_SCM_ cells from negatively selected naive Pan-T cells using EasySep Human Naive Pan T cell Isolation kit (Stemcell technology, #17961) without pre-separating CD4+ or CD8+ T_N_ cells. We believe that these approaches will enable more efficient and reproducible procedures for the manufacturing of highly effective CAR-T cells.

CAR-modified CD8+ T cells harboring T_SCM_-surface phenotype required longer incubation to exert CAR-dependent effector activity compared to the pre-differentiated cells (16 hours *vs* 4 hours, **Figures 3B** and **Supplementary Figure 6B**). The pre-differentiated cells consisted of more differentiated cells, which have potent cytotoxicity than cells harboring T_SCM_ cell phenotype. We also confirmed that 16 hours of incubation was not sufficient to fully induce differentiation of the cells with T_SCM_-surface phenotype to cells with differentiated phenotypes, such as T_CM_ or T_EM_ (**Supplementary Figure 5**). We thus expected the cells derived with our protocol to have less effector activity than those with differentiated phenotypes, and to require longer incubation before acquiring cytotoxicity. These possibilities are currently under investigation.

In recent years, immunotherapy utilizing CAR-engineered T cells has become a highly promising approach, especially for the treatment of blood cancers. T_SCM_ cells have the capacity for both self-renewal and oligopotent differentiation into effector cells upon encounter with antigens; thus, T_SCM_ cells re-directed against their targets could be more effective than mature T cells employed in current clinical trials. Due to the low frequency of T_SCM_ cells in peripheral blood, establishing techniques for efficient expansion with high levels of gene modification will be important for translation to clinical purposes. Our protocol described here is applicable for a prompt implementation of T_SCM_ cell-based immunotherapies against not only cancer but also infectious diseases such as AIDS.

## Data availability statement

The raw data supporting the conclusions of this article will be made available by the authors, without undue reservation.

## Ethics statement

The animal study was reviewed and approved by Guido Eibl, UCLA.

## Author contributions

EK, CJK, wrote the paper. EK, JC, and PYK performed the experiments. ISY, interpreted data. MK designed the research, performed the experiments, analyzed data, interpreted data, and wrote the paper. All authors contributed to the article and approved the submitted version.

## Funding

This work was supported by the California HIV/AIDS Research Grants Program ID13-LA-563 (M.K.), NIH grants R01AI110200 (MK), and RO1 CA232015 (MK). The following reagents were obtained through the NIH AIDS Reagent Program, Division of AIDS, NIAID, NIH: BCBL-1 (Drs. Michael McGrath and Don Ganem), Jurkat-ΔKS and Jurkat-HXBC2 (4) (Dr. Joseph Sodroski), and TF228.1.16 (Drs. Zdenka Jonak and Steve Trulli). Maestro 2 multispectral imaging system was performed at the CNSI Advanced Light Microscopy/Spectroscopy Shared Resource Facility at UCLA, supported by NIH-NCRR shared resources grant (CJX1-443835-WS-29646) and NSF Major Research Instrumentation grant (CHE-0722519).

## Conflict of interest

The authors declare that the research was conducted in the absence of any commercial or financial relationships that could be construed as a potential conflict of interest.

## Publisher’s note

All claims expressed in this article are solely those of the authors and do not necessarily represent those of their affiliated organizations, or those of the publisher, the editors and the reviewers. Any product that may be evaluated in this article, or claim that may be made by its manufacturer, is not guaranteed or endorsed by the publisher.

## Abbreviations

T_SCM_, stem cell memory T; CAR, chimeric antigen receptor; HIV-1, human immunodeficiency virus type 1.
