# Supplementary material for: Efficient derivation of chimeric-antigen receptor-modified TSCM cells
Source: Front Immunol. 2022 Jul 28;13:877682. doi: 10.3389/fimmu.2022.877682 (PMC9366550; doi:10.3389/fimmu.2022.877682)
Supplement: Supplementary file 1 [file DataSheet_1.docx]

**Supplementary tables:**

|  | Gattinoni *et al*. | Cieri *et al*. |
| --- | --- | --- |
| Initial PBMCs | 200 x10^6^  (100%) | 200 x10^6^  (100%) |
| CD8+ T_N_ | 13.0±2.3 x10^6^  (6.50%) | 13.0±2.3 x10^6^  (6.50%) |
| Total cell numbers | 46.7±12.1 x10^6^  (23.4%) | 96.0±23.5 x10^6^  (48.0%) |
| T_N_ | 1.94±2.51 x10^6^  (0.97%) | 11.7±6.50 x10^6^  (5.85%) |
| T_SCM_ | 0.41±0.22 x10^6^  (0.21%) | 1.88±1.2 x10^6^  (0.94%) |
| T_CM_ | 1.22±2.1 x10^6^  (0.61%) | 8.09±3.33 x10^6^  (4.05%) |
| T_EM_ | 6.95±2.35 x10^6^  (3.48%) | 3.00±2.64 x 10^6^  (1.50%) |

**Supplementary Table 1. Comparison of two published procedures for derivation of CD8+ T cells harboring T_SCM_-surface phenotype.**

CD8+ T_N_ cells were obtained from freshly isolated PBMCs (200 x 10^6^) and stimulated for 2 days with Dynabeads Human T-Activator CD3/CD28 (Thermofisher) by following the instructions in the presence (Gattinoni *et al.*) or absence (Cieri *et al.*) of 300 IU/mL IL-2 and 5 µM TWS119. The cells were cultured for additional 12 days using conditions described by either Gattinoni *et al* (1). or Cieri *et al* (2). Cells with the surface phenotypes for T_N,_ T_SCM_, T_CM_, and T_EM_ were sorted by the gating strategy as shown in Supplementary Figure 2. Numbers shown in parentheses are % populations against the initial PBMC numbers. “Total cell numbers” show numbers recovered following 12 days culture after 2-day stimulation. Experiments were repeated 3 times and obtained similar results.

|  | **No antibody** | **0.5 µg/mL**  **anti-CD3** | **1.0 µg/mL**  **anti-CD3** | **0.5 µg/mL anti-CD3 + CD28** | **1.0 µg/mL anti-CD3 + CD28** |
| --- | --- | --- | --- | --- | --- |
| **Initial PBMCs** | 200 x 10^6^ (100%) | 200 x 10^6^ (100%) | 200 x 10^6^ (100%) | 200 x 10^6^ (100%) | 200 x 10^6^ (100%) |
| **Total EGFP+ cell number** | 2.54 x 10^6^ (1.27%) | 35.0 x 10^6^ (17.51%) | 46.1 x 10^6^ (23.05%) | 51.3 x 10^6^ (25.64%) | 45.5 x 10^6^ (22.75%) |
| **EGFP+ T_N_** | 1.46 x 10^6^ (0.73%) | 7.54 x 10^6^ (3.77%) | 6.64 x 10^6^ (3.58%) | 5.52 x 10^6^ (2.76%) | 2.78 x 10^6^ (1.39%) |
| **EGFP+ T_SCM_** | 0.77 x 10^6^ (0.39%) | 7.35 x 10^6^ (3.68%) | 7.84 x 10^6^ (3.92%) | 5.61 x 10^6^ (2.80%) | 3.77 x 10^6^ (1.89%) |
| **EGFP+ T_CM_** | 0.01 x 10^6^ (0.01%) | 0.11 x 10^6^ (0.06%) | 0.11 x 10^6^ (0.06%) | 0.23 x 10^6^ (0.11%) | 0.19 x 10^6^ (0.09%) |
| **EGFP+ T_EM_** | 0.03 x 10^6^ (0.02%) | 1.44 x 10^6^ (0.72%) | 1.08 x 10^6^ (0.54%) | 0. 94 x 10^6^ (0.47%) | 1.24 x 10^6^ (0.62%) |
| **EGFP+ T_EMRA_** | 0.02 x 10^6^ (0.01%) | 1.01 x 10^6^ (0.51%) | 0.59 x 10^6^ (0.29%) | 0.39 x 10^6^ (0.19%) | 0.65 x 10^6^ (0.32%) |

**Supplementary Table 2. Comparison of different stimulation conditions for derivation of EGFP marked-CD8+ T cells harboring T_SCM_-surface phenotype.**

The average numbers from each population in EGFP-positive cells in Figure 2B were summarized. Numbers shown in parentheses are % populations against the initial PBMC numbers. Total EGFP+ cell numbers show the total EGFP+ cell numbers recovered following 28 days of culture. Anti-CD28 antibody was used at 2 µg/mL.

**Supplementary Figures:**

**Supplementary Figure 1**


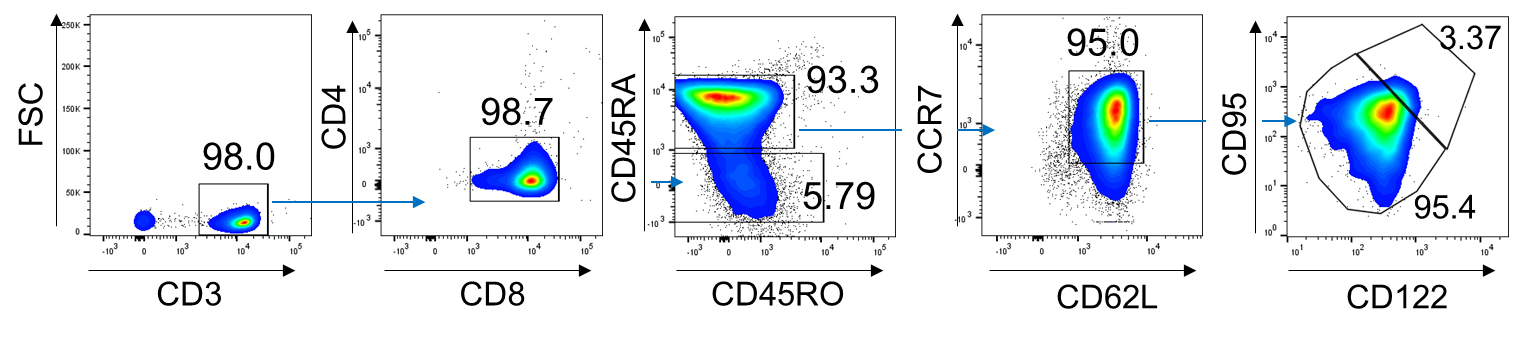


**Supplementary Figure 1**. **Post-sort analysis of CD8+ T_N_ cell population**.

Freshly isolated human CD8+ T_N_ cells were stained with anti-CD3, CD4, CD8, CD45RA, CD45RO, CCR7, CD62L, CD95 and CD122 antibodies and analyzed by flow cytometry. Experiments were repeated more than 5 times with similar results. One representative experiment is shown.


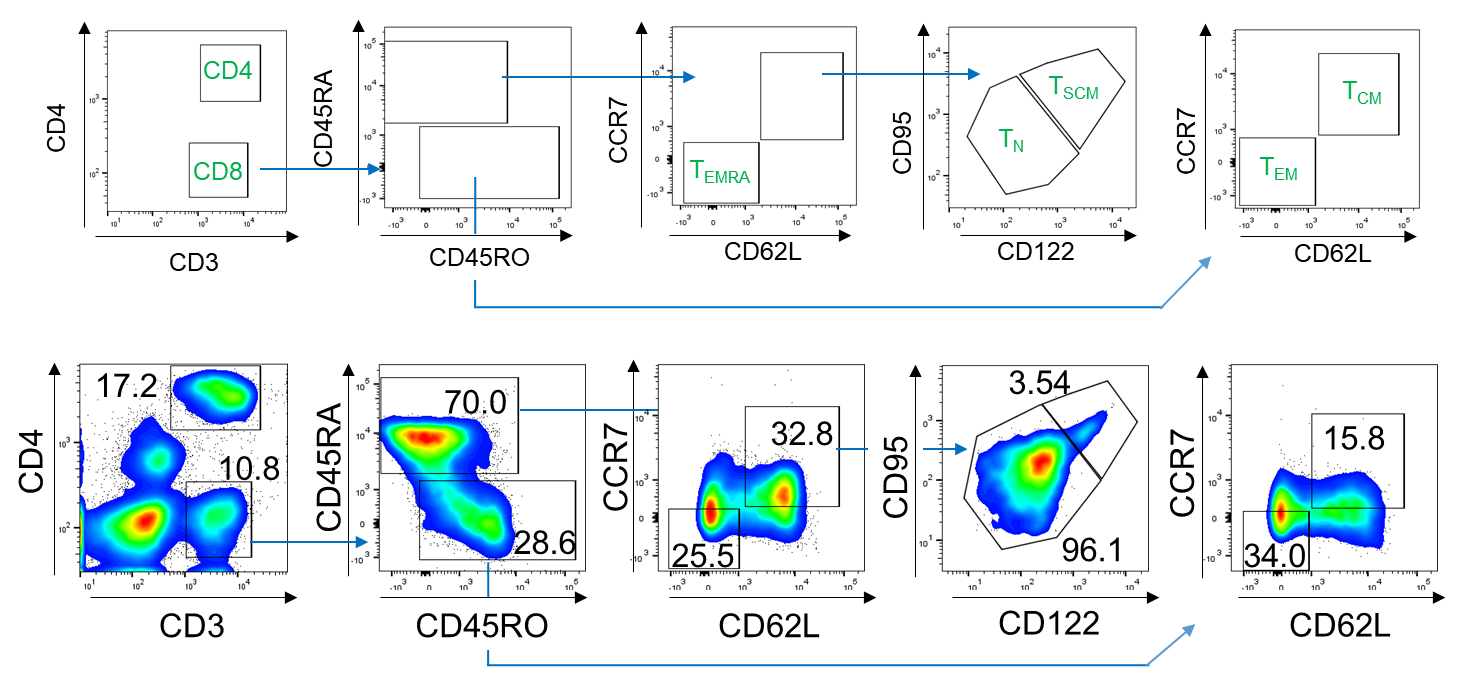


**Supplementary Figure 2**. **Gating strategy for each T cell population.**

Freshly isolated human PBMCs were stained with anti-CD3, CD4, CD45RA, CD45RO, CCR7, CD62L, CD95 and CD122 antibodies, and cell-surface profiles were analyzed by flow cytometry. Naïve T (T_N_): CD45RA+/CD45RO-/CD62L+/CCR7+/CD122^low^/CD95^low^, Stem cell memory T (T_SCM_): CD45RA+/CD45RO-/CD62L+/CCR7+/CD122^high^/CD95^high^, Central memory T (T_CM_): CD45RA-/CD45RO+/CD62L+/CCR7+, Effector memory T (T_EM_): CD45RA-/CD45RO+/CD62L-/CCR7-, Terminally differentiated effector memory RA T (T_EMRA_): CD45RA+/CD45RO-/CD62L-/CCR7-. Experiments were repeated 3 times with similar results. One representative experiment is shown.

**
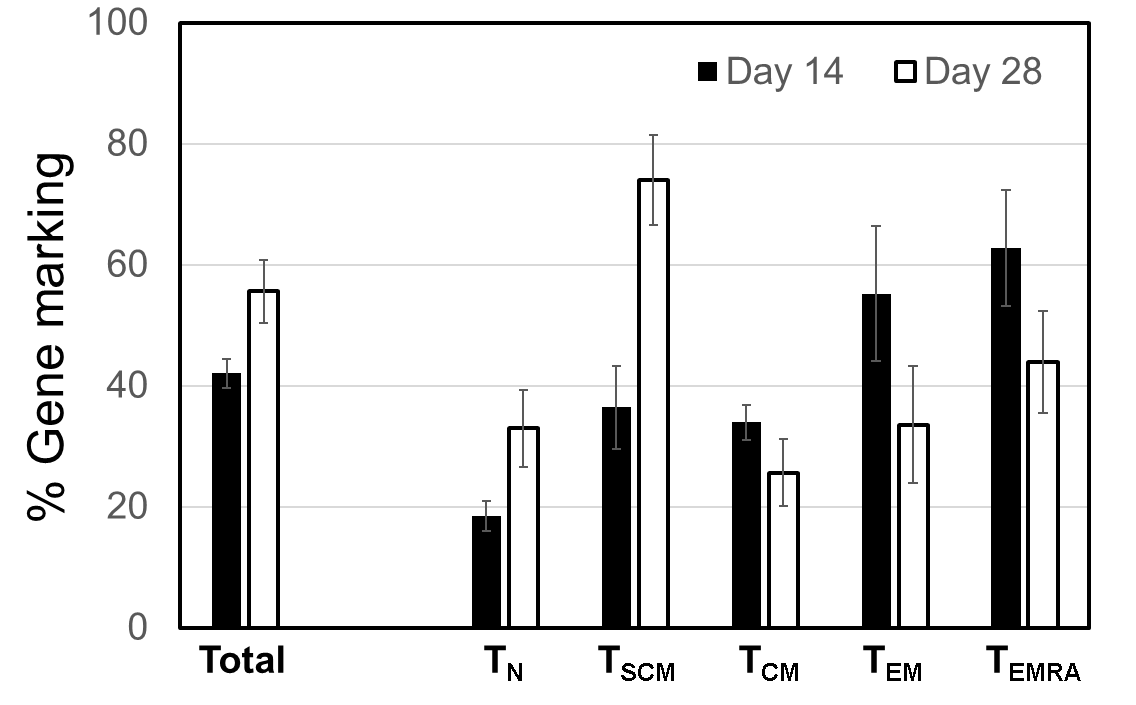
**

**Supplementary Figure 3. % Gene-marking in T_SCM_ cell population increases over the course of culture.**

CD8+ T_N_ cells were transduced with lentiviral vector encoding EGFP following stimulation with soluble anti-CD3 antibody at 0.5 µg/mL for 2 days and cultured in the presence of 5 ng/mL of IL-7 and IL-15 as shown in Figure 1A. The cells were stained for CD45RA, CD45RO, CCR7, CD62L, CD95 and CD122, and analyzed on BD LSRFortessa at day14 (left) and day28 (right). % Gene marking of total cells and cells with T_N_, T_SCM_, T_CM_, T_EM_, and T_EMRA_ phenotypes were plotted.

**
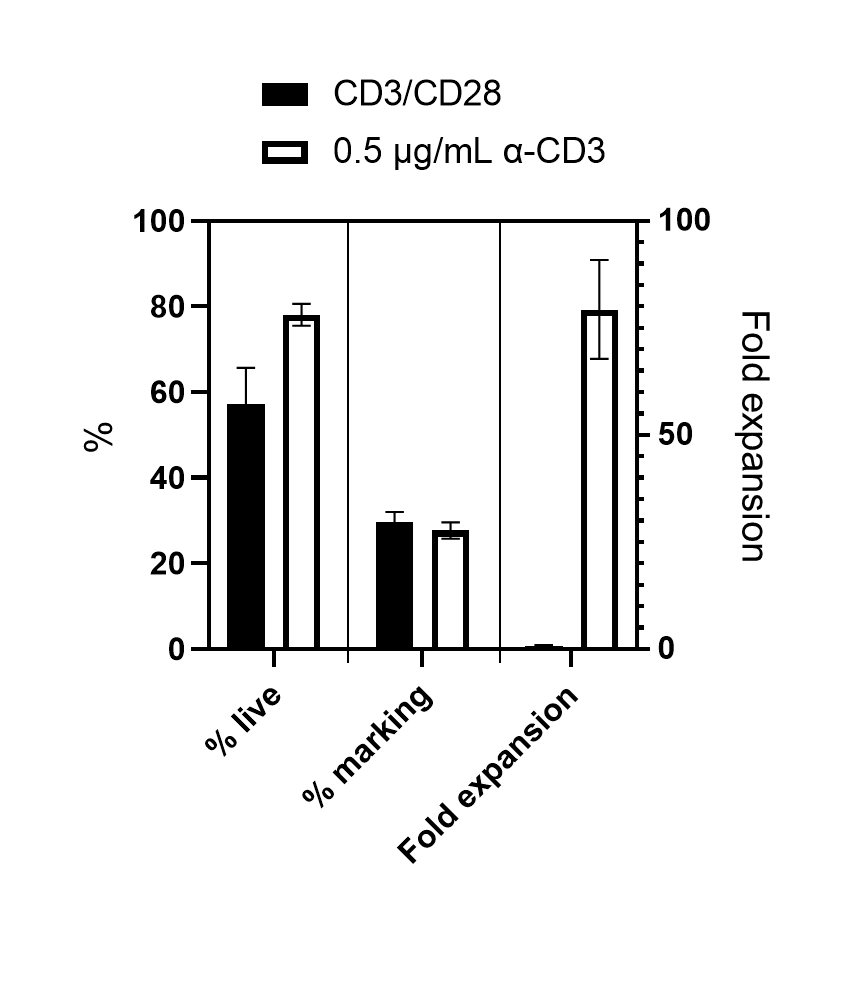
Supplementary Figure 4. Poor fold T-cell expansion by CD3/CD28 beads stimulation.**

CD8+ T_N_ cells were stimulation with soluble anti-CD3 antibody at 0.5 µg/mL or CD3/CD28 coated Dynabeads (ThermoFisher, Pittsburgh, PA) for 2 days following with an instruction. Cells were transduced with a lentiviral vector encoding EGFP and further cultured in the presence of 5 ng/mL of IL-7 and IL-15 as shown in Figure 1A or 100 IU/mL of human IL-2 (R&D systems, Minneapolis, MN) for additional 26 days. % Live cell was counted on TC-10 (Bio-Rad, Hercules, CA). % Gene marking of total cells were analyzed on LSRFortessa. Fold expansion was calculated based on the initial cell number used for stimulation and final cell number following total 28 days culture. Experiments were performed with three biological replicates.

**
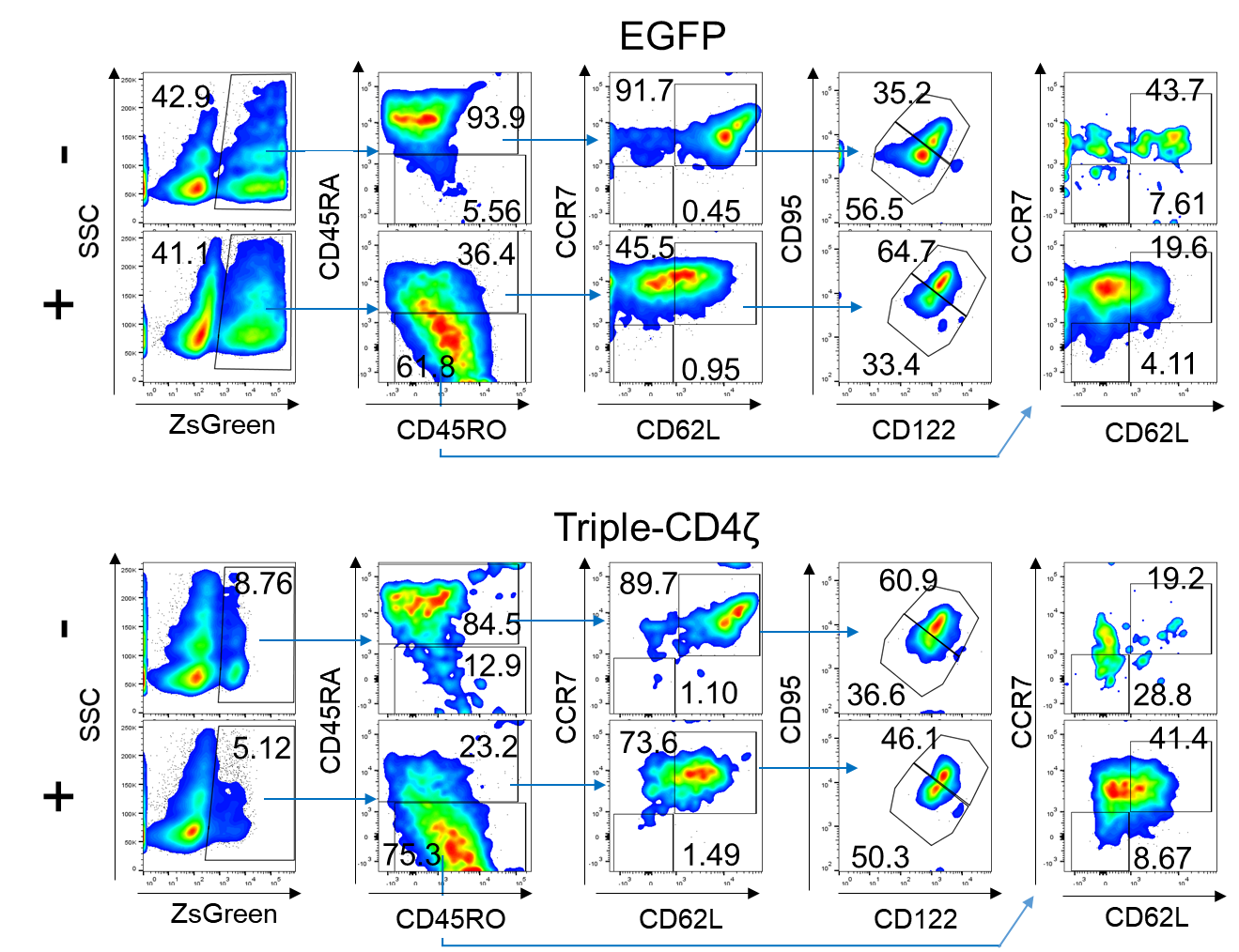
**

**Supplementary Figure 5**.  **Derivation of CD8+ T_SCM_ cells engineered with Triple-CD4ζ and validation of their oligopotency.**

Freshly isolated CD8+ T_N_ cells were stimulated for 2 days with 0.5 µg/mL of anti-CD3 antibody in T-cell medium, followed by transduction with a lentiviral vector encoding Triple-CD4ζ or EGFP as a control. The cells were then cultured in the presence of 5 ng/mL of IL-7 and IL-15 for an additional 12 days. Half of the cells were induced to differentiate by co-stimulating with 0.5 µg/mL of anti-CD3 and 2.0 µg/mL of CD28 antibodies for 2 days and cultured for an additional 12 days in the presence of 5 ng/mL of IL-7 and IL-15. The remaining half of the cells were cultured in the presence of 5 ng/mL of IL-7 and IL-15 without stimulation (-). Cell surface marker profiles for T_N_, T_SCM_, T_CM_, T_EM,_ and T_EMRA_ were analyzed by flow cytometry. Experiments were repeated 3 times with similar results. One representative experiment is shown.

**
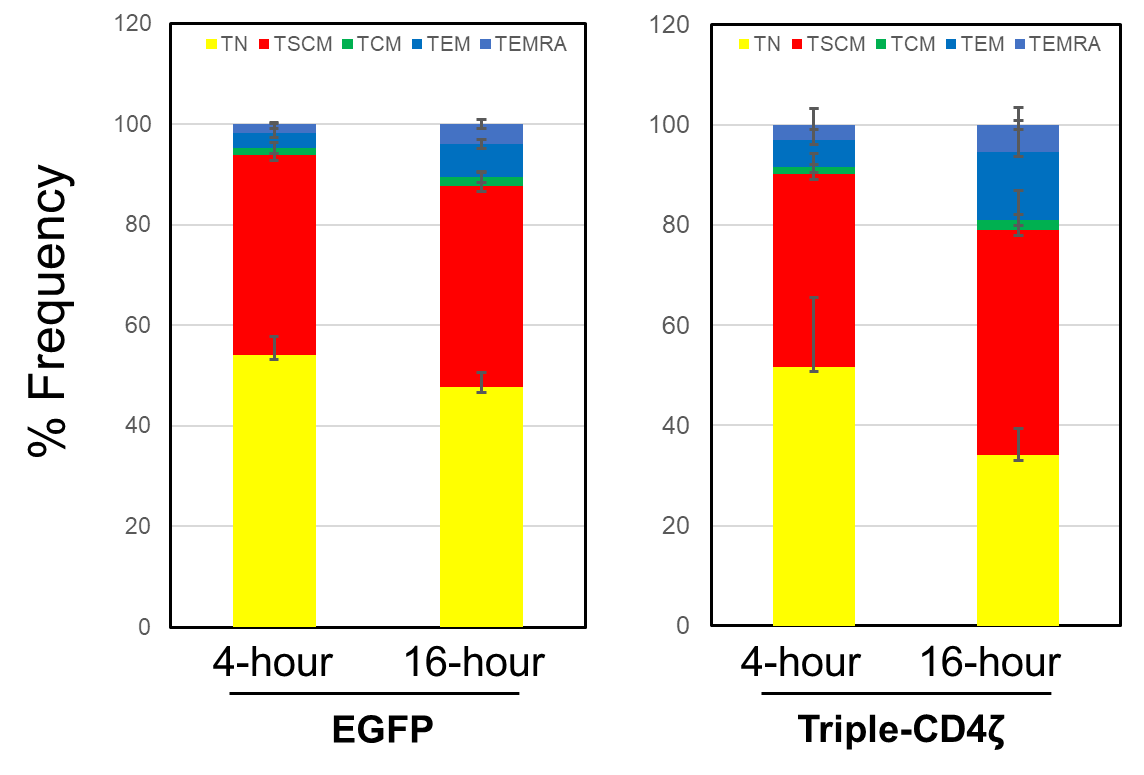
**

**Supplementary Figure 6. Short antigen stimulation is insufficient to induce surface phenotypic changes in Triple-CD4ζ-modified CD8+ T cells.**

Triple-CD4ζ or EGFP-modified CD8+ T cells (0.5 x 10^6^) were cocultured with the same number of mCherry-labeled Jurkat cells constitutively expressing HIV-1_HXBC2_ envelope protein (HXBC2, target cells) for 4- or 16-hours. Cells were stained for CD45RA, CD45RO, CCR7, CD62L, CD95 and CD122, and surface phenotypes of gene-marked cells were analyzed by flow cytometry. % Frequency of gene-marked cells with T_N_, T_SCM_, T_CM_, T_EM_, and T_EMRA_ phenotypes was plotted. Experiments were repeated three times. Error bars show the standard deviation of a data set.

**
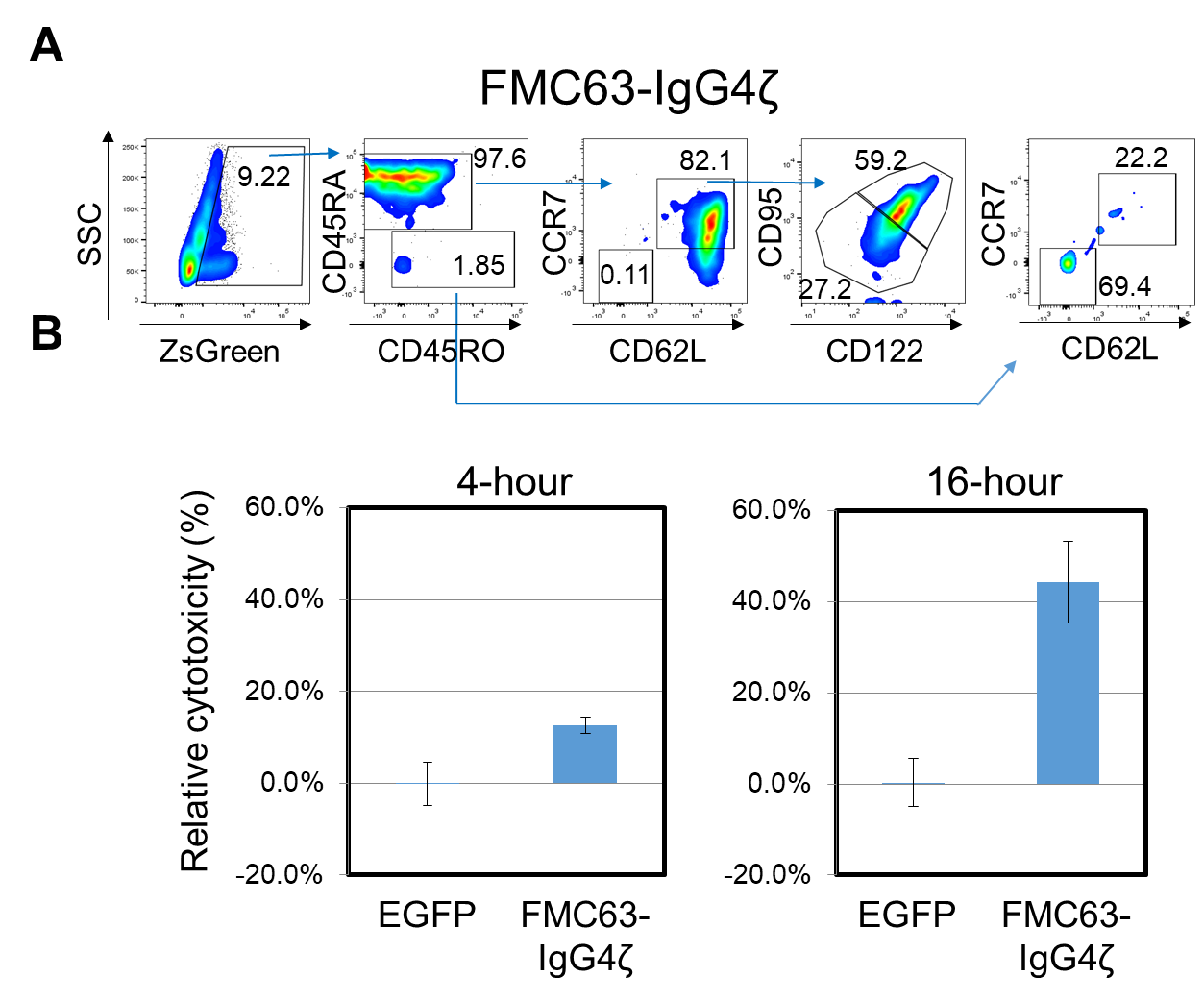
**

**Supplementary Figure 7. Derivation of CD8+ T_SCM_-cells engineered with anti-CD19 CAR, FMC63-IgG4ζ and validation of their effector activity.**

**A.** Freshly isolated human CD8+ T_N_ cells were stimulated for 2 days with 0.5 µg/mL of anti-CD3 antibody in T-cell medium, followed by transduction with a lentiviral vector encoding anti-CD19 CAR (FMC63-IgG4ζ). The cells were cultured in the presence of 5 ng/mL of IL-7 and IL-15 for an additional 26 days (total 28 days post-1^st^ stimulation), and cell-surface marker profiles for T_N_, T_SCM_, T_CM_, T_EM,_ and T_EMRA_ were analyzed by flow cytometry. Experiments were repeated 3 times with similar results. One representative experiment is shown. **B.** The gene-marked cells were plated at 5 x 10^4^ cells/100 µL in a 96-well plate and co-incubated with the same numbers of TagBFP-labeled CD19- non-Hodgkin lymphoma B cell line (BCBL-1, non-target control) and mStrawberry-labeled CD19+ Burkitt lymphoma line (Ramos, target cells) for 4 or 16 hours. Total numbers of each cell type were determined by MACSQuant and relative killing of target cells relative to non-target cells was determined. Experiments were repeated 3 times with similar results. Cytotoxicity assays were performed in biological triplicate.


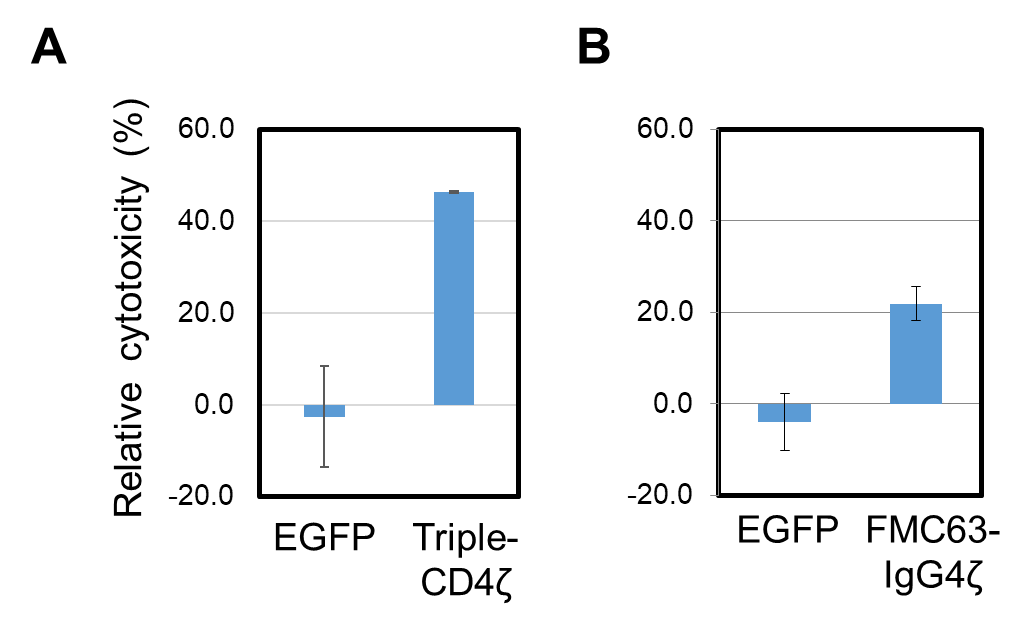


**Supplementary Figure 8. CAR-dependent cytotoxicity of CD8+ T_SCM_ cells modified with Triple-CD4ζ or FMC63-IgG4ζ.**

Freshly isolated human CD8+ T_N_ cells were modified with a lentiviral vector encoding Triple-CD4ζ (**A**) or FMC63-IgG4ζ (**B**) following two days stimulation with 0.5 µg/mL of anti-CD3 antibody. After 26 days of culture in the presence of 5 ng/mL of IL-7 and IL-15, the cells were plated at 5 x 10^4^ cells/100 µL in a 96-well plate and co-incubated with the same numbers of mStrawberry-labeled TF228.1.16 (target cell) constitutively expressing HIV-1_BH10_ envelope protein and TagBFP-labeled Jurkat cells (ΔKS) (**A.** non-target control for Triple-CD4ζ CAR) or BCBL-1 cells (**B.** non-target control for FMC63-IgG4ζ CAR) for 16 hours. Total numbers of each cell were determined by MACSQuant and relative cytotoxicity of target cells relative to non-target cells was determined.

**References:**

1 L. Gattinoni, E. Lugli, Y. Ji, Z. Pos, C. Paulos, M. Quigley, J. Almeida, E. Gostick, Z. Yu, C. Carpenito, E. Wang, D. Douek, D. Price, C. June, F. Marincola, M. Roederer, and N. Restifo, A human memory T cell subset with stem cell-like properties. *Nature Medicine* 17 (2011) 1290-7.

2 N. Cieri, B. Camisa, F. Cocchiarella, M. Forcato, G. Oliveira, E. Provasi, A. Bondanza, C. Bordignon, J. Peccatori, F. Ciceri, M. Lupo-Stanghellini, F. Mavilio, A. Mondino, S. Bicciato, A. Recchia, and C. Bonini, IL-7 and IL-15 instruct the generation of human memory stem T cells from naive precursors. *Blood* 121 (2013) 573-84.
